# Turbidity and fecal indicator bacteria in recreational marine waters increase following the 2018 Woolsey Fire

**DOI:** 10.1038/s41598-022-05945-x

**Published:** 2022-02-14

**Authors:** Marisol Cira, Anisha Bafna, Christine M. Lee, Yuwei Kong, Benjamin Holt, Luke Ginger, Kerry Cawse-Nicholson, Lucy Rieves, Jennifer A. Jay

**Affiliations:** 1 Department of Civil and Environmental Engineering, University of California, Los Angeles, Los Angeles, CA USA; 2 Jet Propulsion Laboratory, California Institute of Technology, Pasadena, CA USA; 3 Department of Earth and Environmental Science, University of Pennsylvania, Philadelphia, PA USA; 4 Heal the Bay, Santa Monica, CA USA

**Keywords:** Environmental sciences, Natural hazards, Ocean sciences, Environmental microbiology

## Abstract

Wildfires increase runoff and sediment yields that impact downstream ecosystems. While the effects of wildfire on stream water quality are well documented, oceanic responses to wildfire remain poorly understood. Therefore, this study investigated oceanic responses to the 2018 Woolsey Fire using satellite remote sensing and in situ data analyses. We examined 2016–2020 turbidity plume (n = 192) and 2008–2020 fecal indicator bacteria (FIB, n = 15,015) measurements at variable proximity to the Woolsey Fire. Shifts in coastal water quality were more pronounced in the “inside” region, which drained the burn area. The inside region experienced 2018–2019 plume surface area monthly means that were 10 and 9 times greater than 2016–2017 and 2017–2018 monthly means, respectively. Further, linear regressions showed that 2018–2019 three-day precipitation totals produced plumes of greater surface area. We also noted statistically significant increases in the inside region in 2018–2019 total coliform and *Enterococcus* monthly means that were 9 and 53 times greater than 2008–2018 monthly means, respectively. These results indicate that sediment and microbial inputs to coastal ecosystems can increase substantially post-wildfire at levels relevant to public and environmental health, and underscore the benefit of considering remote sensing and in situ measurements for water quality monitoring.

## Introduction

In the western United States, wildfire activity has increased since the late twentieth century^1,2^ in frequency, duration, and season length^3,4^. Wildfire activity is projected to surge in the second half of the twenty-first century in response to future climate changes^2^. In Southern California, where wildfire activity is driven by precipitation and aridity^5–7^, and wildfire growth is driven by Santa Ana winds^8–10^, a projected reduction in precipitation^11^, accretion in aridity^12^, and seasonal shift in Santa Ana wind events^13^ are expected to exacerbate wildfire conditions.

As wildfire activity increases, soil hydrology and, by extension, water quality will increasingly become affected. More specifically, wildfire accelerates soil erosion rates by removing vegetation and litter cover, intensifying and translocating soil hydrophobicity, and inducing soil sealing^14–20^. During and immediately after a wildfire, soil erosion begins as dry ravel. At the onset of rainfall, runoff is unable to infiltrate burned soils, resulting in soil erosion via rill networks and debris flows^21^. Ultimately, these processes produce heightened runoff and sediment yields^16,17,22^ that mobilize and transport contaminants to downstream ecosystems.

Wildfires have been shown to alter the physical and chemical water quality of receiving streams. For example, elevated turbidity levels in stream waters post-wildfire are well documented^23–31^. However, wildfire impacts on microbial water quality remain elusive^32^. Most notably, water quality responses to wildfire in receiving oceans have been overlooked^33^. Additionally, studies have been largely limited by sparse sampling, hindering broad spatial and temporal observations^34^.

Satellite remote sensing can be used to help resolve gaps in spatial and temporal sampling of water quality to help evaluate coastal conditions following wildfire events. Water quality variables, such as turbidity, can be derived using optical data acquired through remote sensing platforms including Landsat and Sentinel-2. A semi-empirical algorithm for turbidity^35^ has been applied and evaluated in water bodies across the world of varying optical complexities^36,37^. Good agreement between satellite-derived and in situ turbidity measurements indicate that satellite imagery can be used as a complement to on-going monitoring programs that include collection and analysis of water samples, with the potential to expand spatial and temporal coverage across regions and decades. This study focuses on the remote sensing data processing and interpretation, and relies on previous validation studies^35–37^. Nonetheless, this study did show consistency between satellite-derived turbidity and coincident in situ light transmissivity measurements (Supplementary Text S1, Supplementary Fig. S1).

The goal of this study was to investigate spatial and temporal shifts in coastal water quality associated with the 2018 Woolsey Fire. We analyze long-term and high-frequency turbidity and fecal indicator bacteria (FIB) datasets to examine water quality before, during, and after the Woolsey Fire. We hypothesize that increases in monthly mean sediment and FIB levels will be associated with beaches draining the fire burn area, compared with beaches adjacent to or outside the fire burn area.

## Data and methods

### Study area

The Santa Monica Mountains are located in the Southwestern United States. The Santa Monica Mountains are part of the east–west trending Transverse Ranges of Southern California. The dominant vegetation communities, chaparral and coastal sage scrub, are adapted to the Mediterranean climate conditions of wet winters, dry summers, and frequent fires^38^.

Despite temperatures in the mid-70°Fs, the Santa Monica Mountains faced extreme fire weather conditions on November 8, 2018. At 10 am local time, relative humidity dropped to 5% and wind gusts soared to 35 mph. At 2 pm, a powerline failure near the Santa Susana Field Lab ignited the Woolsey Fire^39^, and the Santa Ana winds, Southern California foehn winds, pushed the fire south into the Santa Monica Mountains. The Woolsey Fire burned nearly 100,000 acres and destroyed over 1600 structures in the Ventura and Los Angeles counties before being fully contained on November 21, 2018^40^. Post-fire, the California Watershed Emergency Response Team used hydrological models to assess flood and debris flow risks, and their watershed modeling approximated a two to fivefold increase in post-fire flows^40^.

**Figure 1:**
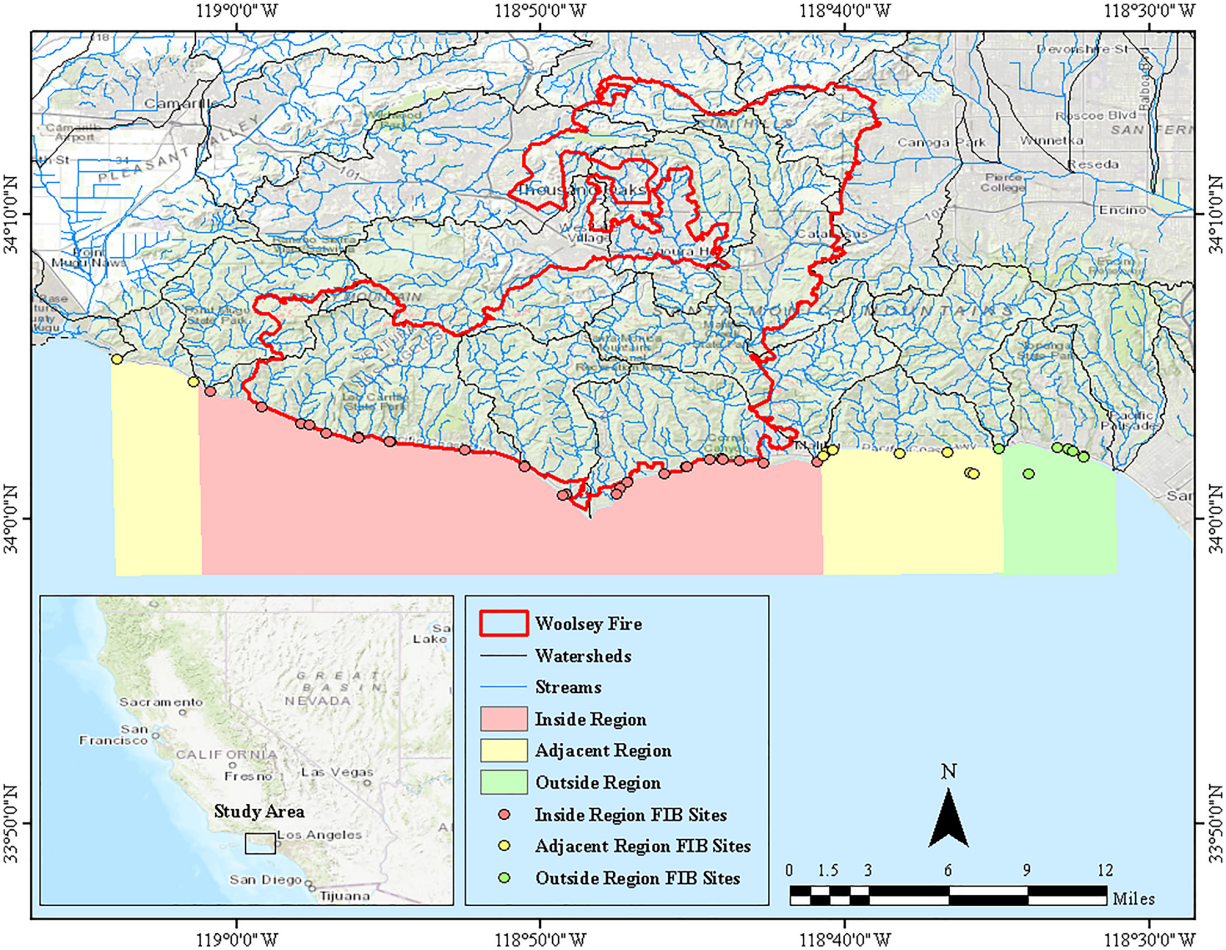
The study area map shows the Woolsey Fire (red lines), watersheds (black lines), and streams (blue lines) in the Santa Monica Mountains coastal range. The map also indicates the location of the 26 FIB sites (red circles) in the inside region (red), 10 FIB sites (yellow circles) in the adjacent region (yellow), and 8 FIB sites (green circles) in the outside region (green).

To study the impact of increased flow and sediment delivery to the coast, we divided our study area into three regions using the Woolsey Fire and watershed boundaries (Fig. 1). The “inside” region received discharges from burned watersheds. The “adjacent” region, immediately to the west and east of the Woolsey Fire, received runoff primarily from unburned watersheds that were adjacent to burned watersheds. The “outside” region, to the east of the Woolsey Fire, received flows from unburned watersheds. Unlike the adjacent region, we did not define an outside region to the west of the Woolsey Fire due to a difference in land use and land cover. Namely, this region received runoff primarily from agriculture and salt marsh, while the rest of our study area received runoff primarily from chaparral and coastal sage scrub^38^. The coordinates of these regions can be found in Supplementary Table S1.

### Sentinel-2 image acquisition and processing

We used the Sentinel-2 imagery Level-1C product T11SLT tile available to users via EarthExplorer (https://earthexplorer.usgs.gov/). We obtained 48 cloud-free Sentinel-2 images spanning from August 2016 to April 2020. The European Space Agency’s Copernicus Sentinel-2 mission utilizes a constellation of two identical satellites, in the same orbit 180° apart, to provide a high revisit time. Sentinel-2A was launched on June 23, 2015, and Sentinel-2B was launched on March 7, 2017. Sentinel-2 images land and coastal areas with a wide swath high-resolution multispectral imager. Sentinel-2 revisit time, spatial resolution, swath width, and spectral bands are listed in Supplementary Table S2.

Sentinel-2 imagery, for the study area and each region of the study area, were atmospherically corrected and processed in ACOLITE, an open-source software downloadable from GitHub (https://github.com/acolite/acolite)^36,37^. The image outputs were visualized in ArcGIS.

ACOLITE uses a dark spectrum fitting algorithm and an exponential extrapolation algorithm to atmospherically correct images^41,42^. ACOLITE derives turbidity, in Formazine Nephelometric Units (FNU), from water surface reflectance as shown in Eq. (1):

1 $$T= \frac{A {\rho }_{w}}{1- \frac{{\rho }_{w}}{C}},$$

where ${\rho }_{w}$ is the water-leaving radiance reflectance, and A and C are band-specific calibration coefficients. This study uses the Dogliotti et al. algorithm in ACOLITE, which uses the calibration coefficients corresponding to the red band (645 nm) when ${\rho }_{w}$(645 nm) < 0.05 and the near-infrared band (859 nm) when ${\rho }_{w}$ (645 nm) > 0.07. When 0.05 < ${\rho }_{w}$ (645 nm) < 0.07, the Dogliotti et al. algorithm uses a linear weighting function to calculate turbidity.

Assessing the detection limit of the Dogliotti et al. algorithm was not within the scope of this study given that it has been validated in optically diverse waters^36,37^. However, false positives were of concern due to clouds, fog, and wind. Therefore, we screened manually for clouds, utilized a cloud mask, and flagged for outliers to minimize any false positives.

### Turbidity threshold and plume surface area calculations

We developed an automated plume detection algorithm in Python to characterize an increase in turbidity resulting from the wildfire. To determine the turbidity threshold of our plume detection algorithm, we utilized the November 30, 2018, image of the inside region, which captured discharges from burned watersheds after a rain event. Four 2 km squares were utilized as the regions of interest around discharge points. For each square, 1000 unique points were randomly selected. For each point, we extracted the mean turbidity from a 5 × 5 pixel window. The minimum, lower quartile, mean, median, upper quartile, and maximum values were calculated for all points in each square. The lower quartile, which was 4.6 FNU, was selected as the turbidity threshold. While not shown, we evaluated the probability density functions within plume and non-plume regions and verified that the threshold of 4.6 FNU was consistently higher than background levels (2.3 FNU, considered for 5 scenes), providing confidence that 4.6 FNU was sufficient to minimize any false positives in plume detection.

To compute the plume surface area from an image, we assigned a binary value to each pixel. If a pixel had a turbidity value above the turbidity threshold, then the pixel was assigned a 1, otherwise, the pixel was assigned a 0. The pixels assigned a 1 were counted and converted to surface area. We performed the turbidity threshold and plume surface area calculations in Python. We calculated plume surface area monthly means by region and date range in RStudio.

### Fecal indicator bacteria data

FIB originate from the gastrointestinal tracts of humans and other warm-blooded animals and are therefore used as proxies for fecal contamination. Although they are not typically harmful themselves, FIB are used as pathogen indicators since fecal matter may contain a myriad of disease-causing organisms^43^. Studies have demonstrated that rainstorms increase FIB levels in seawater^44–46^ and that seawater exposure after a rainstorm increases incidence rates of swimming-associated illnesses^47^. Therefore, the California Department of Public Health advises beachgoers to stay out of the water for a minimum of 3 days after a storm event greater than 0.1 inches. Additionally, the State Water Resources Control Board (SWRCB) has set the standards for recreational marine waters at 10,000 colony-forming units (CFU)/100 mL for total coliform (TC) and 104 CFU/100 mL for *Enterococcus* (ENT)^48^.

To assess the effects of wildfire on bacterial water quality, we accessed FIB data from July 2008 to June 2020 for the Los Angeles County and Ventura County coast from SWRCB (https://www.waterboards.ca.gov/water_issues/programs/beaches/search_beach_mon.html). The FIB data included TC and ENT measurements in most probable number (MPN)/100 mL. For this study area, measurements were taken at 26 sites in the inside region (n = 8978), 10 sites in the adjacent region (n = 2758), and 8 sites in the outside region (n = 3279) (Fig. 1). For the sites in Los Angeles County, measurements were taken at a minimum of once a week throughout the year. However, for the sites in Ventura County, measurements were only taken at a minimum of once a week from April to October. In RStudio, monthly means and standard errors were calculated for TC and ENT by region, date range, and weather condition.

### Precipitation data

We acquired daily precipitation data from the NOAA National Climatic Data Center (https://www.ncdc.noaa.gov), for the Los Angeles International Airport, from June 2008 to June 2020. We used RStudio to calculate 3-day and monthly precipitation totals. The 3-day precipitation totals were used to categorize our FIB data by weather condition. FIB data with a 3-day precipitation total greater than 0.1 inches was categorized as wet weather data, otherwise, it was categorized as dry weather data^49^. We also computed the means and standard errors of monthly precipitation totals by date range, where possible. Date ranges were ordered by rainfall season, which runs from July 1 through June 30. The 2016–2020 and 2008–2020 monthly precipitation totals are shown in Supplementary Fig. S2.

### Statistics

We used histograms and quantile–quantile (Q–Q) plots to test whether the FIB data were normal. The data were found to be non-normal. Therefore, two-sided Wilcoxon rank-sum tests with continuity correction were used to compare 2008–2018 to 2018–2019 by month, region, and weather condition.

We also performed Pearson correlations and linear regressions for all variables. A multiple linear regression using 3-day precipitation totals, region, and fire year (2018–2019) and non-fire years (2016–2017, 2017–2018, and 2019–2020) to predict plume surface area was further examined. Residual diagnostics were evaluated to check the validity of the assumptions made when fitting the multiple linear regression model. Linear regressions of plume surface area and 3-day precipitation totals were visualized by region and fire year and non-fire years. A sensitivity analysis was also performed by removing data points with 3-day precipitation totals equal to 0 to test the influence of these data points. All statistical computations were performed in RStudio.

### Systematic literature review

We conducted a systematic review on March 12, 2021, using Web of Science. The following three searches were performed: (1) “wildfire” and “turbidity” (41 results, 11 retained), (2) “wildfire” and “total coliforms OR fecal coliforms OR Escherichia coli OR Enterococcus” (13 results, 1 retained), (3) “wildfire,” “water quality,” and “remote sensing” (9 results, 1 retained). Studies that did not directly investigate the impacts of wildfire in the Western United States were removed. Retained studies are summarized in Supplementary Table S3. The current study is novel in that it is the first to investigate the impacts of wildfire on ocean turbidity and FIB. In addition, it is the second to apply remote sensing and in situ techniques to study the effects of wildfire on coastal water quality.

## Results

### Remote sensing analysis: turbidity and plume surface area

**Figure 2:**
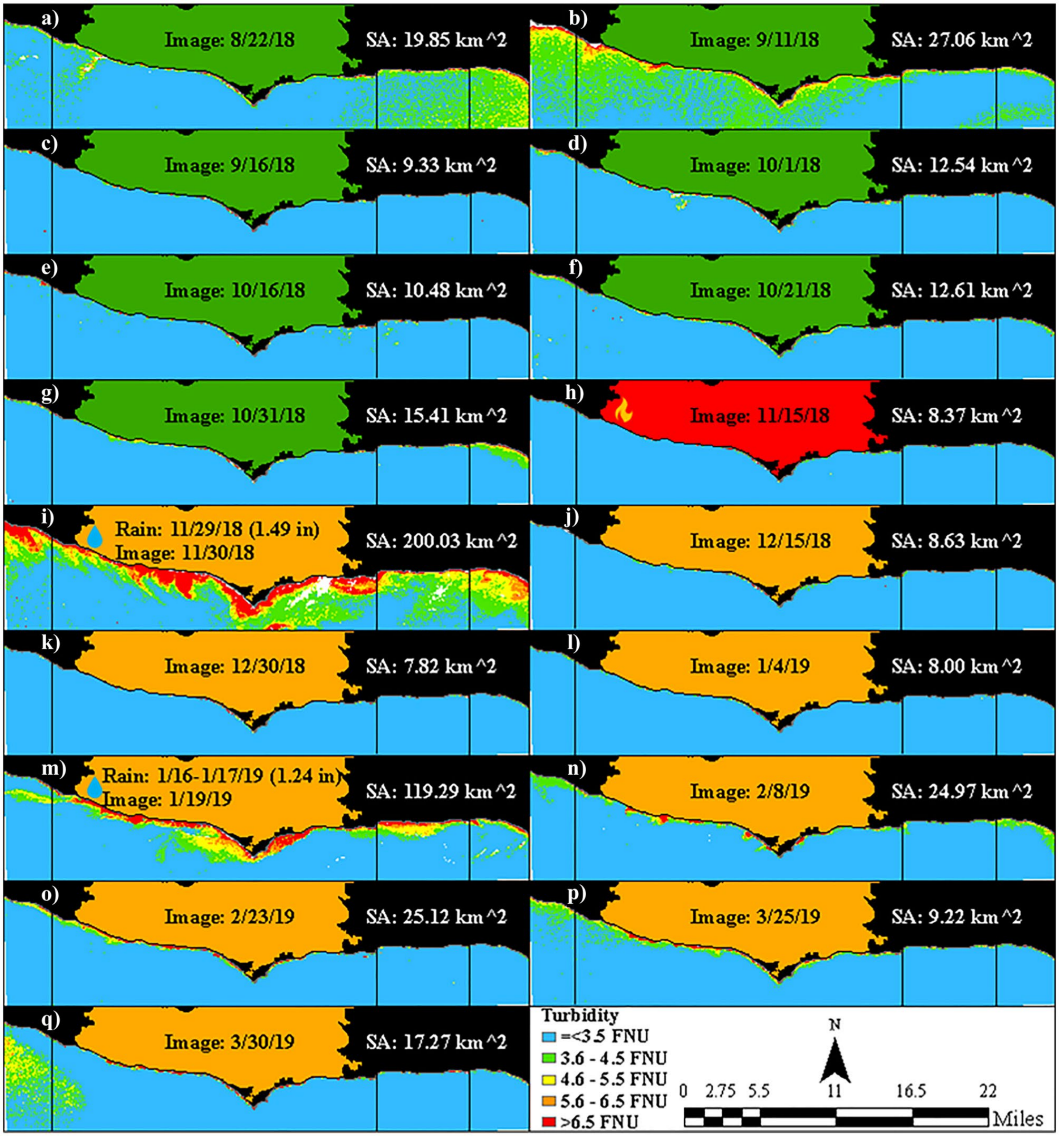
Imagery of the study area from 2018 to 2019, where available, with turbidity and plume surface area (SA). (**a–g**) Pre-fire images, (**h**) active Woolsey Fire (November 8–21, 2018) image, (**i**–**q**) post-fire images, and (**i**,**m**) images within three days of a rain event. Vertical black lines correspond to region boundaries in Fig. 1. Post-fire rain events produced up to 200 km^2^ plumes (exceeding the 4.6 FNU threshold).

The remote sensing analysis revealed notable changes in physical water quality in response to the 2018 Woolsey Fire. Imagery of the study area from 2018 to 2019 (Fig. 2) indicated an increase in plume intensity and extent in post-fire images (Fig. 2i–q), in comparison to pre-fire images (Fig. 2a–g). Generally, more turbidity values in the 4.6 to greater than 6.5 FNU range were observed post-fire. More specifically, the surface area of plumes exceeding the 4.6 FNU threshold shifted from 9 to 27 km^2^ pre-fire to 8 to 200 km^2^ post-fire. These changes in turbidity were mostly attributed to post-fire rain events (Fig. 2i,m).

**Figure 3:**
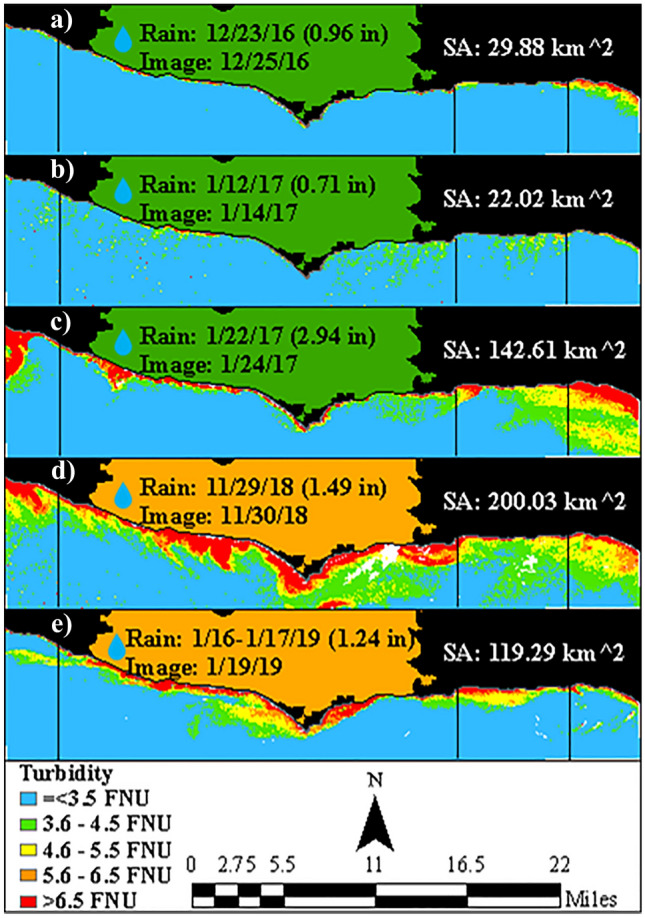
Imagery within three days of a rain event greater than 0.7 inches, where possible, with turbidity and plume surface area (SA). (**a**–**c**) Pre-fire rain events and (**d**,**e**) post-fire rain events. There were no cloud-free images within three days of a rain event greater than 0.7 inches in 2020. Vertical black lines correspond to region boundaries in Fig. 1. Rain events from 2016 to 2019 show post-fire rain events produced plumes (exceeding the 4.6 FNU threshold) of greater extent and intensity.

Imagery of similar rain events from 2016 to 2019 further illustrated that the turbidity from post-fire rain events was anomalous (Fig. 3d,e), compared to pre-fire rain events (Fig. 3a–c). For example, a previous rain event of 2.94 inches (Fig. 3c) produced a smaller plume than a post-fire rain event of 1.49 inches (Fig. 3d). Moreover, while images of previous rain events indicated that regions closest to urban areas were normally more impacted, images of post-fire rain events indicated that the region below the Woolsey Fire was more impacted.

**Figure 4:**
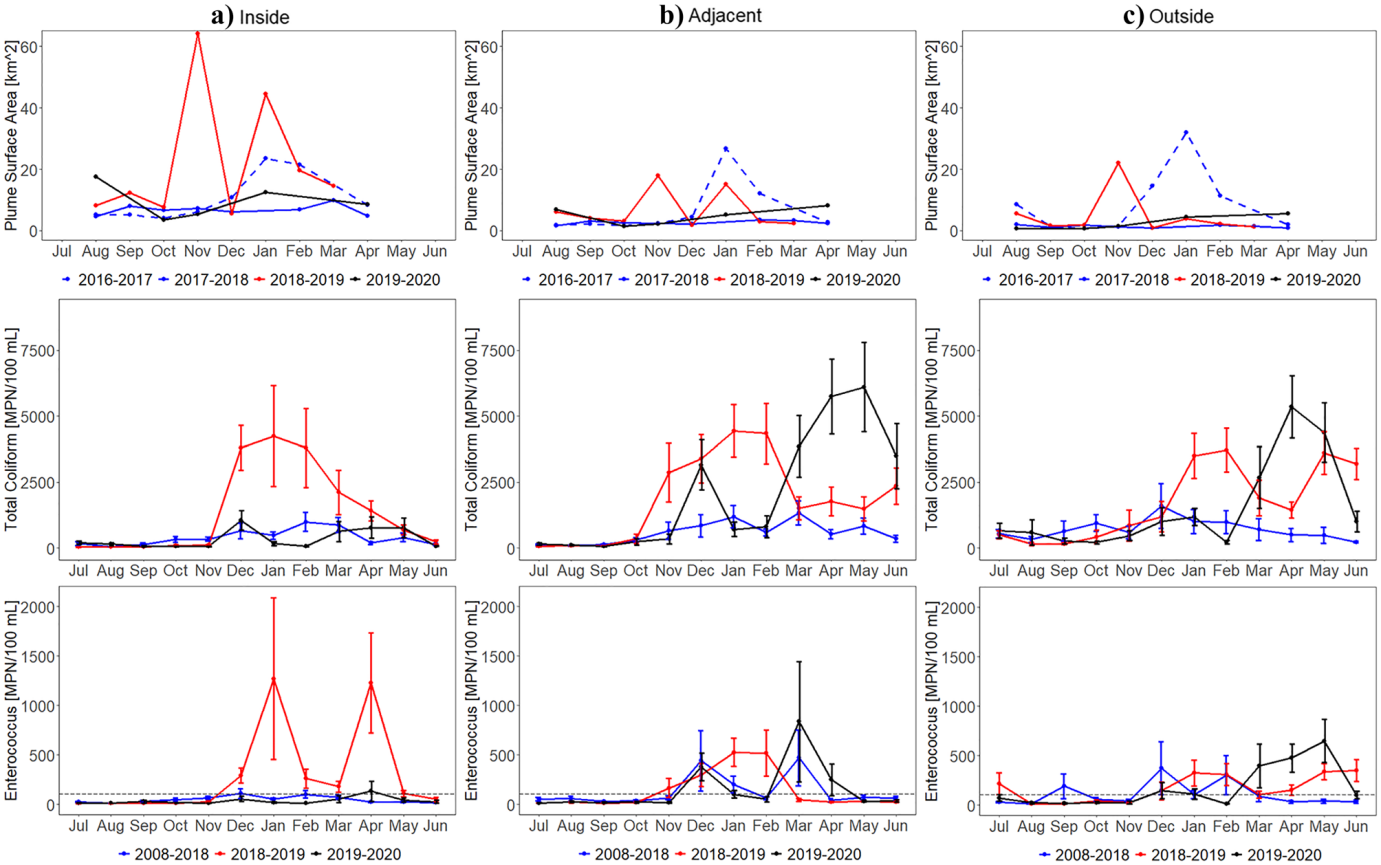
Plume surface area (exceeding the 4.6 FNU threshold, 2016–2020) and FIB (TC and ENT, 2008–2020) monthly means and standard errors for the (**a**) inside, (**b**) adjacent, and (**c**) outside region. The SWRCB standards are indicated with a black dotted line. Please see Fig. 1 for region locations. Plume surface area, TC, and ENT increase in 2018–2019 following the Woolsey Fire (November 8–21, 2018), particularly in the inside region.

Plume surface area monthly means from 2016 to 2020 by region further confirmed that the 2018–2019 plume extent was atypical, particularly for the inside region, which received surface runoff from burned watersheds (Fig. 4). Prior to the Woolsey Fire, the 2018–2019 plume surface area monthly means were similar to those observed in other years for all regions. However, in November, the 2018–2019 plume surface area monthly average increased to 64, 18, and 22 km^2^ in the inside region, adjacent region, and outside region, respectively. In January, the 2018–2019 plume surface area monthly mean also increased in all regions, however, only the inside region’s 45 km^2^ plume differed from those observed in other years.

### In situ analysis: fecal indicator bacteria

The in situ analysis also showed microbial water quality responses to the wildfire. FIB monthly means from 2008 to 2020 by region showed marked increases post-fire, primarily in the inside region, which drained the burned area (Fig. 4). Before the Woolsey Fire, the 2018–2019 TC monthly means were comparable to those observed in other years for all regions. However, in December, the 2018–2019 TC monthly average in the inside region increased and remained elevated through March. Compared to 2008–2018 TC monthly means in the inside region, these elevations were statistically significant in December (W = 43, Z =  − 3.16, p = 0.002, r = 0.47) and statistically highly significant in January (W = 79, Z =  − 3.90, p < 0.001, r = 0.47), February (W = 62, Z =  − 4.06, p < 0.001, r = 0.51), and March (W = 148, Z =  − 3.92, p < 0.001, r = 0.46) (Supplementary Table S4). The 2018–2019 TC monthly average was also discernable between other years in the adjacent region in November (W = 221, Z =  − 2.92, p = 0.003, r = 0.38), January (W = 274, Z =  − 4.19, p < 0.001, r = 0.48), and February (W = 203, Z =  − 4.04, p < 0.001, r = 0.49) and in the outside region in January (W = 251, Z =  − 4.22, p < 0.001, r = 0.49), February (W = 157, Z =  − 4.73, p < 0.001, r = 0.57), and June (W = 113, Z =  − 6.04, p < 0.001, r = 0.67). ENT displayed similar behavior as TC. Prior to the Woolsey Fire, the 2018–2019 ENT monthly means were similar to those observed in other years in all three regions. In December, the 2018–2019 ENT monthly mean average in the inside region also increased and remained elevated through May. In comparison to 2008–2018 ENT monthly means in the inside region, these elevations were statistically significant in December (W = 57, Z =  − 2.75, p = 0.006, r = 0.40), January (W = 143, Z =  − 2.84, p = 0.004, r = 0.34), and February (W = 117, Z =  − 3.06, p = 0.002, r = 0.39) and statistically highly significant in March (W = 174, Z =  − 3.56, p < 0.001, r = 0.42), April (W = 164, Z =  − 5.94, p < 0.001, r = 0.58), and May (W = 161, Z =  − 4.94, p < 0.001, r = 0.51). The 2018–2019 ENT monthly means were also distinguishably different from other years in the adjacent region in January (W = 358, Z =  − 3.31, p < 0.001, r = 0.38) and February (W = 294, Z =  − 2.88, p = 0.004, r = 0.35) and in the outside region in January (W = 402, Z =  − 2.55, p = 0.011, r = 0.30) and June (W = 259, Z =  − 4.57, p < 0.001, r = 0.51). While the January and February observations may be partly in response to unusual monthly precipitation totals (Supplementary Fig. S2), results do indicate that they are also in response to the wildfire. Namely, the 2018–2019 ENT monthly average in January in the inside region (1270 MPN/100 mL) was substantially higher than that of the adjacent region (526 MPN/100 mL) and outside region (326 MPN/100 mL). It is also worth noting that the 2019–2020 TC and ENT monthly means in the inside region did not respond to abnormal precipitation totals in December, March, and April as seen in the adjacent and outside regions.

**Figure 5:**
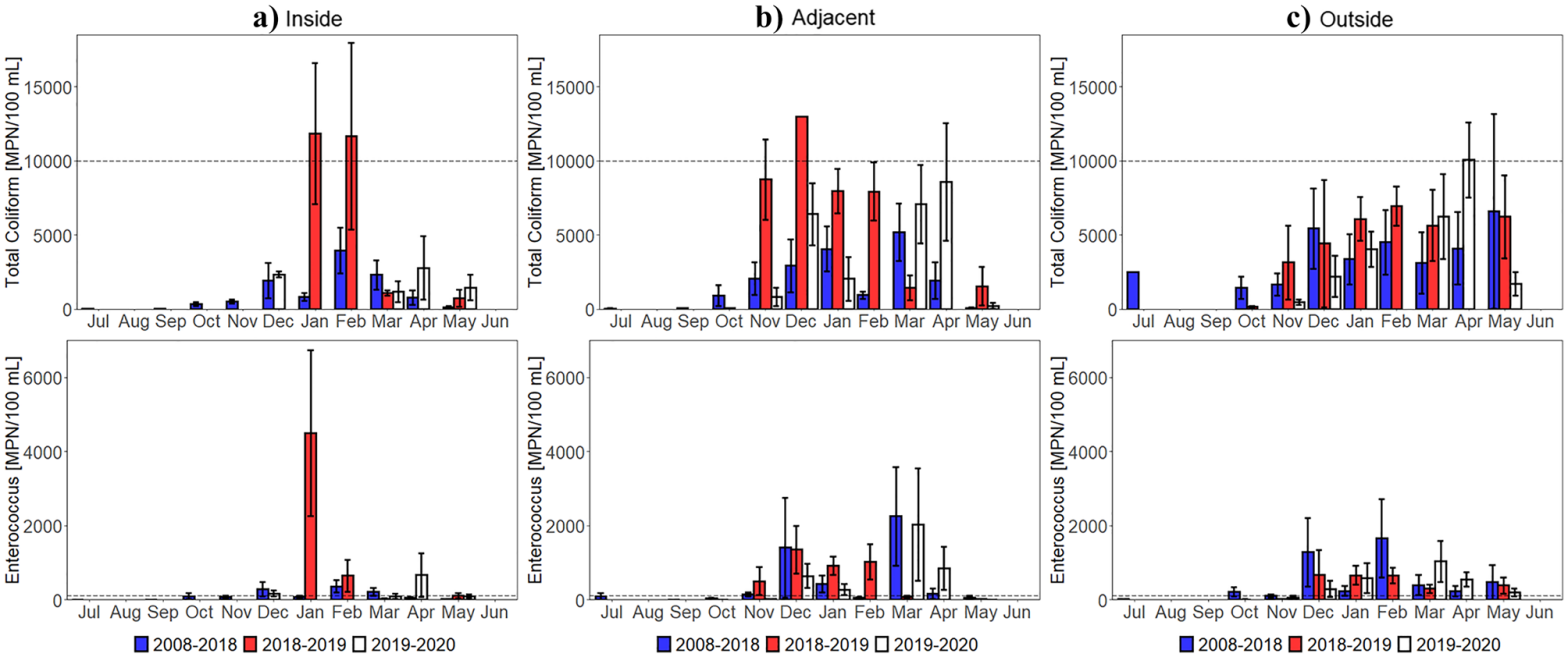
Wet weather FIB (2008–2020) monthly means and standard errors for the (**a**) inside, (**b**) adjacent, and (**c**) outside region. The SWRCB standards are indicated with a black dotted line. Note that the y-axis was changed. Statistics are shown in Supplementary Table S5. Wet weather (3-day precipitation totals greater than 0.1 inches) TC and ENT increase in 2018–2019 following the Woolsey Fire (November 8–21, 2018), more substantially in the inside region.

Examining FIB by weather conditions demonstrated that the shifts in 2018–2019 TC and ENT in the inside region occurred during both wet and dry weather (Supplementary Fig. S3). More precisely, the wet weather 2018–2019 TC monthly means were distinguishably higher than those observed in other years in the inside region in January (W = 3, Z =  − 2.22, p = 0.026, r = 0.55) and in the adjacent region in November (W = 9, Z =  − 2.10, p = 0.035, r = 0.53) and February (W = 15, Z =  − 2.22, p = 0.026, r = 0.52) (Fig. 5, Supplementary Table S5). Figure 5 and Supplementary Table S5 also illustrated that the wet weather 2018–2019 ENT monthly averages were distinctly different in the adjacent region in January (W = 35, Z =  − 2.14, p = 0.032, r = 0.44) and February (W = 17, Z =  − 2.05, p = 0.041, r = 0.48). While there were other highly visible increases in wet weather 2018–2019 TC and ENT monthly means, they were not statistically significant increases (Supplementary Table S5).

**Figure 6:**
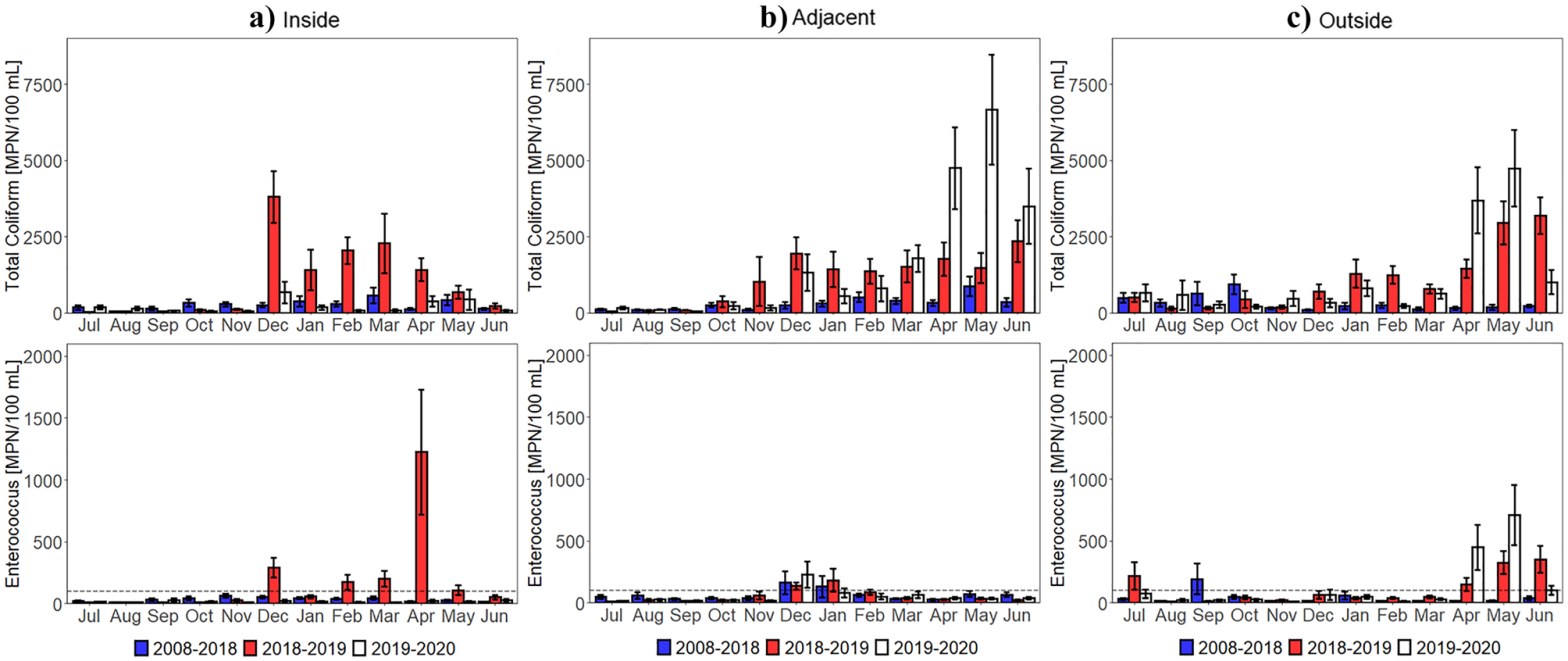
Dry weather FIB (2008–2020) monthly means and standard errors for the (**a**) inside, (**b**) adjacent, and (**c**) outside region. The SWRCB standards are indicated with a black dotted line. Statistics shown in Supplementary Table S6. Dry weather (3-day precipitation totals not greater than 0.1 inches) TC and ENT increase in 2018–2019 following the Woolsey Fire (November 8–21, 2018), more notably in the inside region.

Dry weather 2018–2019 FIB monthly means were more aligned with 2018–2019 FIB monthly means. For instance, the dry weather 2018–2019 TC monthly averages were notably greater than those observed in other years in the inside region in December (W = 25, Z =  − 3.36, p < 0.001, r = 0.55), January (W = 41, Z =  − 3.42, p < 0.001, r = 0.48), February (W = 20, Z =  − 4.18, p < 0.001, r = 0.58), March (W = 69, Z =  − 4.28, p < 0.001, r = 0.55), and April (W = 126, Z =  − 5.96, p < 0.001, r = 0.60), in the adjacent region in November (W = 105, Z =  − 2.91, p = 0.004, r = 0.44) and January (W = 109, Z =  − 3.31, p < 0.001, r = 0.45), and in the outside region in December (W = 100, Z =  − 3.18, p = 0.001, r = 0.48), February (W = 65, Z =  − 3.93, p < 0.001, r = 0.55), and June (W = 113, Z =  − 6.04, p < 0.001, r = 0.67) (Fig. 6, Supplementary Table S6). Furthermore, the dry weather 2018–2019 ENT monthly means were statistically different in the inside region in December (W = 37, Z =  − 2.90, p = 0.004, r = 0.48), February (W = 67, Z =  − 3.01, p = 0.003, r = 0.42), March (W = 76, Z =  − 4.16, p < 0.001, r = 0.53), April (W = 138, Z =  − 5.92, p < 0.001, r = 0.60), and May (W = 134, Z =  − 4.44, p < 0.001, r = 0.48) and in the outside region in February (W = 109, Z =  − 3.25, p = 0.001, r = 0.46), March (W = 200, Z =  − 3.07, p = 0.002, r = 0.40), and June (W = 259, Z =  − 4.57, p < 0.001, r = 0.51). Dry weather 2018–2019 FIB monthly averages also affirmed that the January and February observations from 2018–2019 FIB monthly averages were also in response to the wildfire since they were also derived during dry weather conditions.

### Statistical analysis

**Figure 7:**
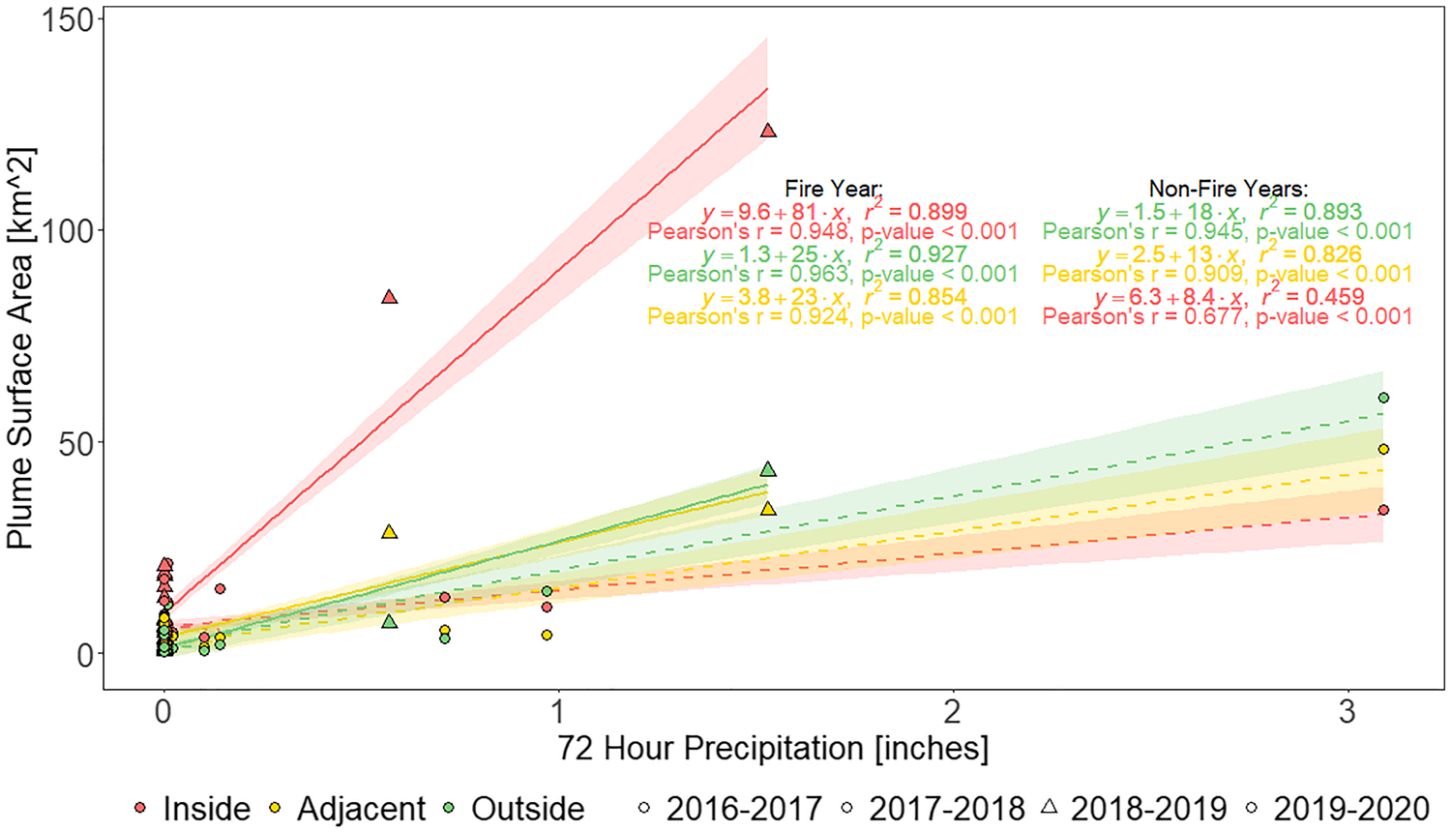
Linear regressions of plume surface area and 3-day precipitation totals for fire year (triangles, solid lines) and non-fire years (circles, dashed lines) in the inside region (red), adjacent region (yellow), and outside region (green) with their respective 95% confidence interval (shaded). Three-day precipitation totals produced plumes (exceeding the 4.6 FNU threshold) with greater surface area during 2018–2019 (fire year), more notably in the inside region.

Moderate Pearson correlations were found between TC and ENT (r = 0.545, p < 0.001), plume surface area and TC (r = 0.547, p < 0.001), and plume surface area and 3-day precipitation totals (r = 0.634, p < 0.001) (Supplementary Table S7). The multiple linear regression used to predict plume surface area based on 3-day precipitation totals, region, and fire year (2018–2019) and non-fire years (2016–2017, 2017–2018, and 2019–2020) was found to be significant (F(4, 285) = 73.82, p < 0.001, R^2^ = 0.509). Examination of individual predictors indicated that 3-day precipitation totals (t = 15.78, p < 0.001), the inside region (t = 4.93, p < 0.001), and the fire year (t = 4.27, p < 0.001) were significant predictors in the model. Residual diagnostics showed that the linearity and normality assumptions made when fitting the multiple linear regression model were valid. In the residuals versus fitted values plot (Supplementary Fig. S4a), residuals were close to and spread along the 0 line with mild departures. In the residuals Q–Q plot (Supplementary Fig. S4b), observations lied along the 45° line with few exceptions. Additionally, all residual normality tests were statistically highly significant (p-value < 0.001) (Supplementary Table S8). Linear regressions of plume surface area and 3-day precipitation totals for the fire year and non-fire years by region confirmed that 2018–2019 post-fire rainstorms produced plumes with greater surface area that were more substantial in the inside region (Fig. 7), as suggested by the satellite imagery. Further, differences between fire year and non-fire years were most notable in the inside region. While the sensitivity analysis (Supplementary Fig. S5) greatly reduced the amount of data, regressions still followed similar trends shown in Fig. 7, indicating that 3-day precipitation totals equal to 0 had little influence on the resulting fit.

## Discussion

Results indicate that the 2018 Woolsey Fire impacted the physical water quality. Imagery of the study area from 2018 to 2019 and of similar rain events from 2016 to 2019 illustrated shifts in plume intensity and extent following post-fire rain events. Plume surface area monthly means from 2016 to 2020 showed that following the wildfire beaches receiving discharges from burned watersheds experienced increases in 2018–2019 plumes in November and January that were up 10 and 9 times greater than 2016–2017 and 2017–2018 plumes, respectively. A multiple linear regression predicting plume surface area revealed that 3-day precipitation totals, the inside region, and the fire year were significant predictors. Further, linear regressions of plume surface area and 3-day precipitation totals by region and fire year and non-fire years demonstrated that post-fire 2018–2019 3-day precipitation totals produced larger plumes, particularly in beaches draining the burn area.

While the effects of wildfire on turbidity in ocean ecosystems have not been previously documented, investigations on the effects of wildfire on turbidity in stream ecosystems have associated heightened turbidity levels to burn severity^29^ and precipitation events^23,25–28,30,31^. In addition, when compared to plumes from urban stormwater runoff in the Santa Monica Bay^50,51^, plumes from burned area surface runoff were greater. This suggests that post-fire runoff is a considerable source of sediment and other debris into ocean ecosystems. These results also suggest that as wildfire activity increases, the Santa Monica Bay (a wildland-urban interface) will experience the cumulative impacts of both wildfire and urbanization. Given that the Transverse Range rivers, which include the Santa Monica Mountain creeks, are responsible for over 75% of the total sediment flux in Southern California^52^, increased wildfire disturbance in this area has the potential to substantially increase sediment delivery to ocean ecosystems.

Results also suggest that the Woolsey Fire degraded the microbial water quality of recreational beaches, and that proximity to wildfire burn areas also plays a role. FIB monthly means from 2016 to 2020 showed that following the Woolsey Fire beaches draining the burned area experienced elevations in 2018–2019 TC from December to March and ENT from December to May that were 9 and 53 times greater than 2008–2018 monthly means, respectively. Results here indicate that post-wildfire runoff and sediments may carry harmful pollutants that may affect beachgoer health.

It is worth noting that FIB may also be delivered to beaches via debris flows. Disastrous debris flows can damage sanitary sewers and septic tanks, contaminating downstream ecosystems with fecal matter. Local agencies may also clear contaminated sediments in public rights-of-way and creek channels and deposit them onto beaches^53^. Fires, floods, and debris flows also have the potential of damaging wastewater treatment plants or deeming them inoperable, which may result in untreated raw sewage being discharged into coastal waters.

Increased sediments may also promote the persistence of FIB. It has been shown that sediments provide nutrients and protection from ultraviolet radiation, temperature fluctuations, and predation^54–59^. However, it is unclear whether burned sediments can affect the survival of FIB in marine waters^32^.

Greater abundance of TC post-wildfire may be due to their widespread occurrence in nature; they are present in plants, soils, and feces from humans and other warm-blooded animals^60^. Greater persistence of ENT post-wildfire may be due to their distinguished ability to survive in salt water^61^. Since ENT are more human-specific^60^ and are more highly associated with gastrointestinal illnesses in marine waters^43,62–65^, dramatic increases in ENT, above the SWRCB standards, are of particular concern. While we did not analyze fecal coliform and *Escherichia coli* (*E.coli*), due to a lack of data, Barron (2020) found that post-fire (2018–2020) mean *E.coli* levels were 10 times greater than pre-fire (2015–2018) mean *E.coli* levels in the surface waters of the North Santa Monica Bay Coastal Watersheds^66^. However, Valenca et al. reported that the growth and persistence of *E. coli* in surface water in the presence of Woolsey Fire residues was less than that in the presence of unburned soil particles. Therefore, future studies should study differences in FIB behavior in different environmental compartments. Future studies could also utilize watershed modeling to help elucidate processes that contribute to observed increases in plumes and FIB.

While FIB are known to increase following rainstorms^44–46^, increases following post-fire rainstorms were greater than what has been observed in the past for TC and ENT during comparable rain events. The beaches draining the burned area exhibited wet weather 2018–2019 TC and ENT monthly means that were up to 14 and 54 times greater than 2008–2018 monthly means, respectively. Further, while Arnold et al. reported wet weather ENT means of approximately up to 300 CFU/100 mL in beaches receiving urban discharges, the current study found wet weather ENT monthly means of up to 4496 MPN/100 mL in beaches receiving runoff and sediment discharges from burned areas.

Due to slow watershed recovery, post-fire sediment yields can remain elevated for 3 months to 10 years^19^. Accordingly, studies have reported short-term^23,24,26,28,30^ and long-term^25,27,29,31^ turbidity increases in streams post-fire. The current study did observe short-term increases in turbidity, within 3 months of the Woolsey Fire. Further, while the TC and ENT responses were more persistent, they also returned to background levels within 6 months of the Woolsey Fire. Since the present study only analyzed a year and a half of post-fire data, future investigations could continue to monitor for any long-term impacts as watersheds recover.

To our knowledge, only one other study has employed remote sensing and in situ techniques to study the effects of wildfire on coastal water quality^67^. This study illustrates how using both satellite and in situ measurements results in a more complete assessment of coastal water quality. For example, with satellite and in situ measurements we were able to evaluate both the physical and microbial water quality. Further, one of the limitations of this study was that we could not obtain in situ FIB measurements for the Ventura County coast from November to March. However, by utilizing satellite turbidity measurements of the Ventura County coast, we were able to gain some indication of the water quality. Similarly, previous studies examining the impacts of wildfire in aquatic ecosystems listed sparse sampling as a limitation that hindered meaningful spatial observations; however, this study’s use of satellite imagery did allow for a supraregional investigation.

This novel approach can be implemented to assess coastal water quality after a major fire event. Results could guide monitoring and public health agencies in sampling and treatment efforts, as well as beach closures. Additionally, researchers could apply this methodology to further investigate the effects of wildfire on coastal water quality. For example, future studies can evaluate oceanic responses to smaller fires or fires that occur more upslope. Future research can also study the effects of post-fire increases in stream nutrients^68–70^ on chlorophyll-a, colored dissolved organic matter, and harmful algal blooms.

There are various remote sensing techniques for detecting plumes. Coastal plumes exhibit differences in turbidity, color, temperature, and salinity from ambient background water that can be observed via multispectral and hyperspectral imagers, thermal infrared (TIR) radiometers, microwave radiometers, and Synthetic Aperture Radar (SAR)^71,72^. In the Southern California Bight, ocean color imagery from SeaWiFS^73^, MODIS^74–76^, and Landsat^77^ have been utilized and combined with radiometers^78,79^ and SAR^50^ to delineate and track plumes.

There does exist some caveats to using satellite imagery. For instance, Sentinel-2 is affected by cloud cover, fog, and wind, and is limited by repeat frequency and time of day of acquisition, making it challenging to study water quality responses to precipitation events. Further, while rapid data processing is possible, real-time image acquisition remains a challenge. Although, efforts are underway to decrease the time for satellite data release to within a day.

In addition, there are challenges to defining turbidity thresholds. Various methods have been used to define a turbidity threshold to delineate plumes. Studies have used an arbitrary value relevant to coral reef health as the turbidity threshold^80^. Studies have also utilized accumulated nLw555 values for 25th and 75th percentile composites^81^, river discharge and plume extension correlation coefficients as a function of threshold values^82,83^, a histogram of the distribution of radiance nLw645^82^, and a finite mixing model^84^ to determine turbidity thresholds.

Based on the results of this study additional research is needed to better understand ecosystem responses to wildfire. Not only is this area of research largely understudied, but the coastal interface is likely to be subjected to numerous climate related threats such as sea level rise and marine heat waves. In addition, we recommend prioritizing low impact development to reduce post-wildfire stormwater and sediment delivery to the coast. However, we also recommend preventative measures for wildfire. In California, most wildfires are caused by human activities, with powerline ignitions representing a substantial proportion of recent wildfire ignition sources^85^. One popular management decision is to de-energize urban areas during times of elevated wildfire risk. However, other options like burying powerlines and replacing aging powerline infrastructure should also be considered. Local governments should also guide development based on wildfire risk^86^. Further, climate change is increasingly creating conditions conducive to wildfire, therefore, a reduction in carbon emissions should also be considered as a strategy for addressing wildfire^87^. Additionally, in Southern California, chaparral is increasingly getting converted to grassland, which poses a higher fire risk^88^. Thus, we should also consider restoring and preserving chaparral landscapes in the wildland-urban interface.

## Supplementary Information

Supplementary Information.

## Data Availability

The data used to support the findings of this study are included within this article and the Supplementary Information.
