# Supplementary material for: Turbidity and fecal indicator bacteria in recreational marine waters increase following the 2018 Woolsey Fire
Source: Sci Rep. 2022 Feb 14;12:2428. doi: 10.1038/s41598-022-05945-x (PMC8844011; doi:10.1038/s41598-022-05945-x)
Supplement: Supplementary file 1 — Supplementary Information. [file 41598_2022_5945_MOESM1_ESM.pdf]

Supplementary Information for

Turbidity and Fecal Indicator Bacteria in Recreational Marine Waters Increase Following the 2018 Woolsey Fire

Marisol Cira[1,2], Anisha Bafna[2,3], Christine M. Lee[2], Yuwei Kong[1], Benjamin Holt[2], Luke Ginger[4], Kerry Cawse-Nicholson[2], Lucy Rieves[4], and Jennifer A. Jay[1]

[1]Department of Civil and Environmental Engineering, University of California, Los Angeles, Los Angeles, CA, USA

[2]Jet Propulsion Laboratory, California Institute of Technology, Pasadena, CA, USA

[3]Department of Earth and Environmental Science, University of Pennsylvania, Philadelphia, PA, USA

[4]Heal the Bay, Santa Monica, CA, USA

Contents of this file

Text S1
Figures S1 to S5
Tables S1 to S8

Text S1. Light Transmission Data

We compared satellite turbidity measurements with *in situ* light transmission measurements provided by LA Sanitation for various stations in the Santa Monica Bay[1]. Light transmission measurements taken within 1.5 hours of a Sentinel-2 overpass, which generally occurred at 11:45 am local time, were selected (Bailey and Werdell, 2006). The mean turbidity from a 5x5 pixel window of each station was extracted. Results are presented in Fig. S1.

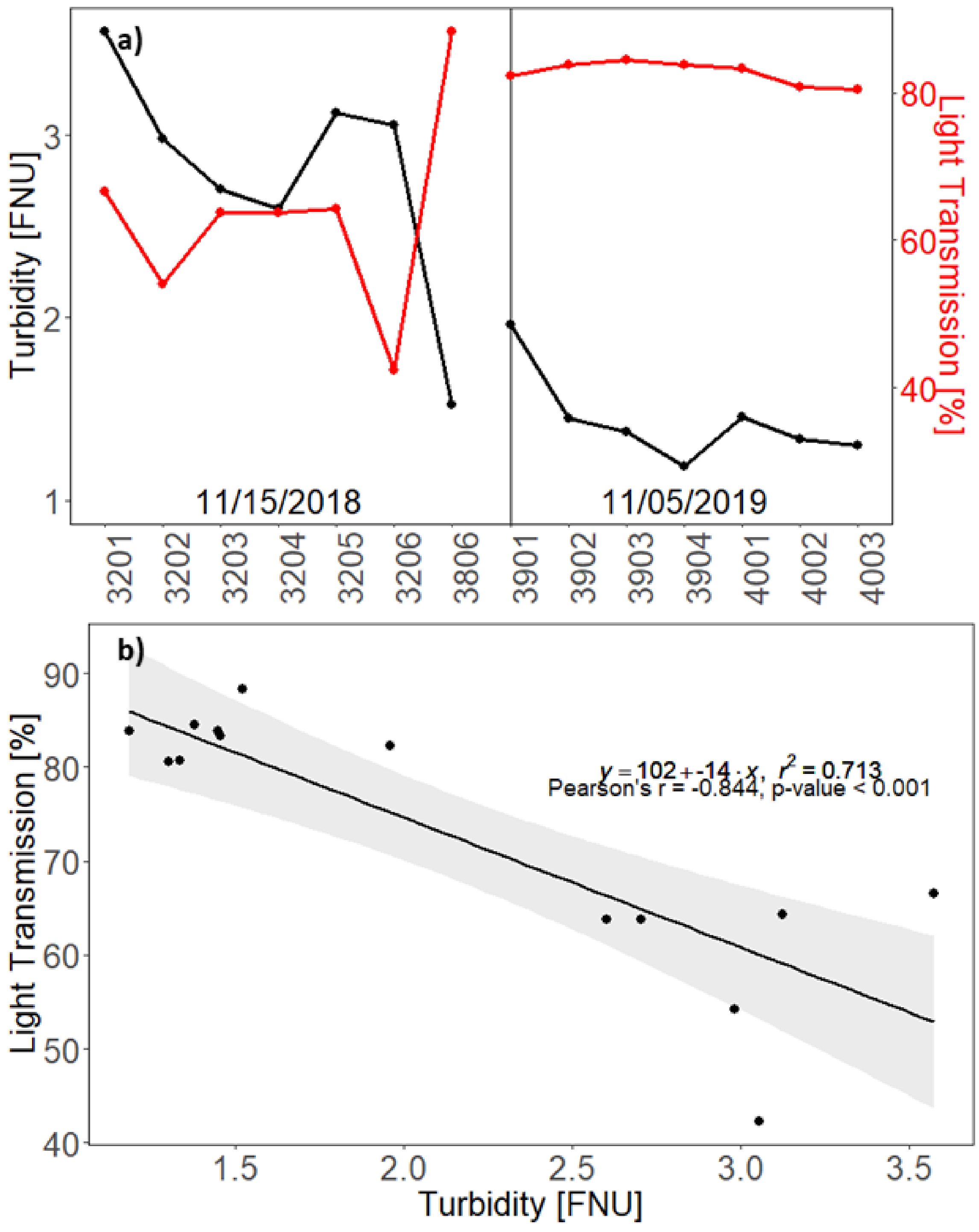

Figure S1. Turbidity and light transmission (**a**) matchups and (**b**) linear regression with 95% confidence interval (shaded).

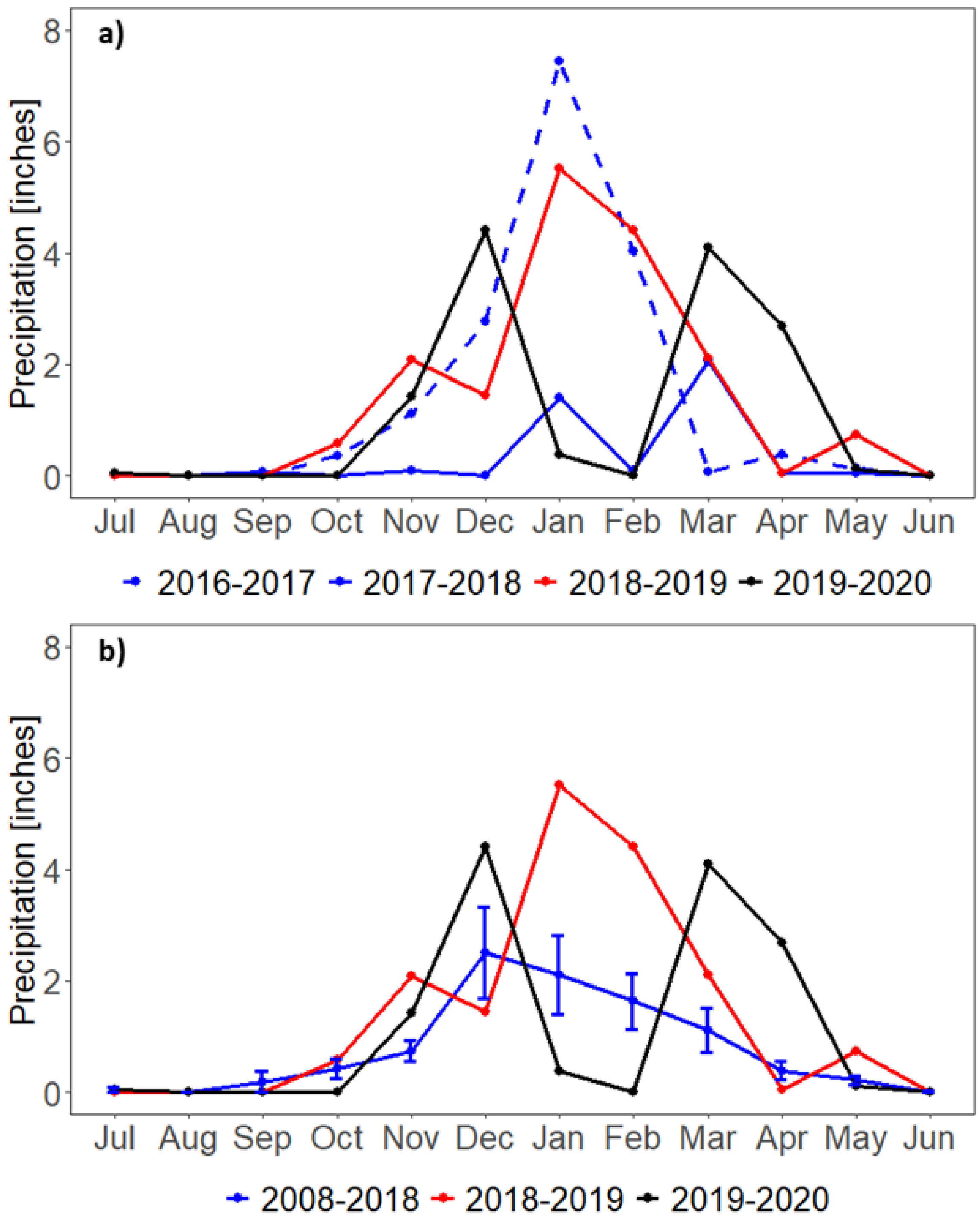

Figure S2. Monthly precipitation totals for (**a**) 2016-2020 and (**b**) 2008-2020.

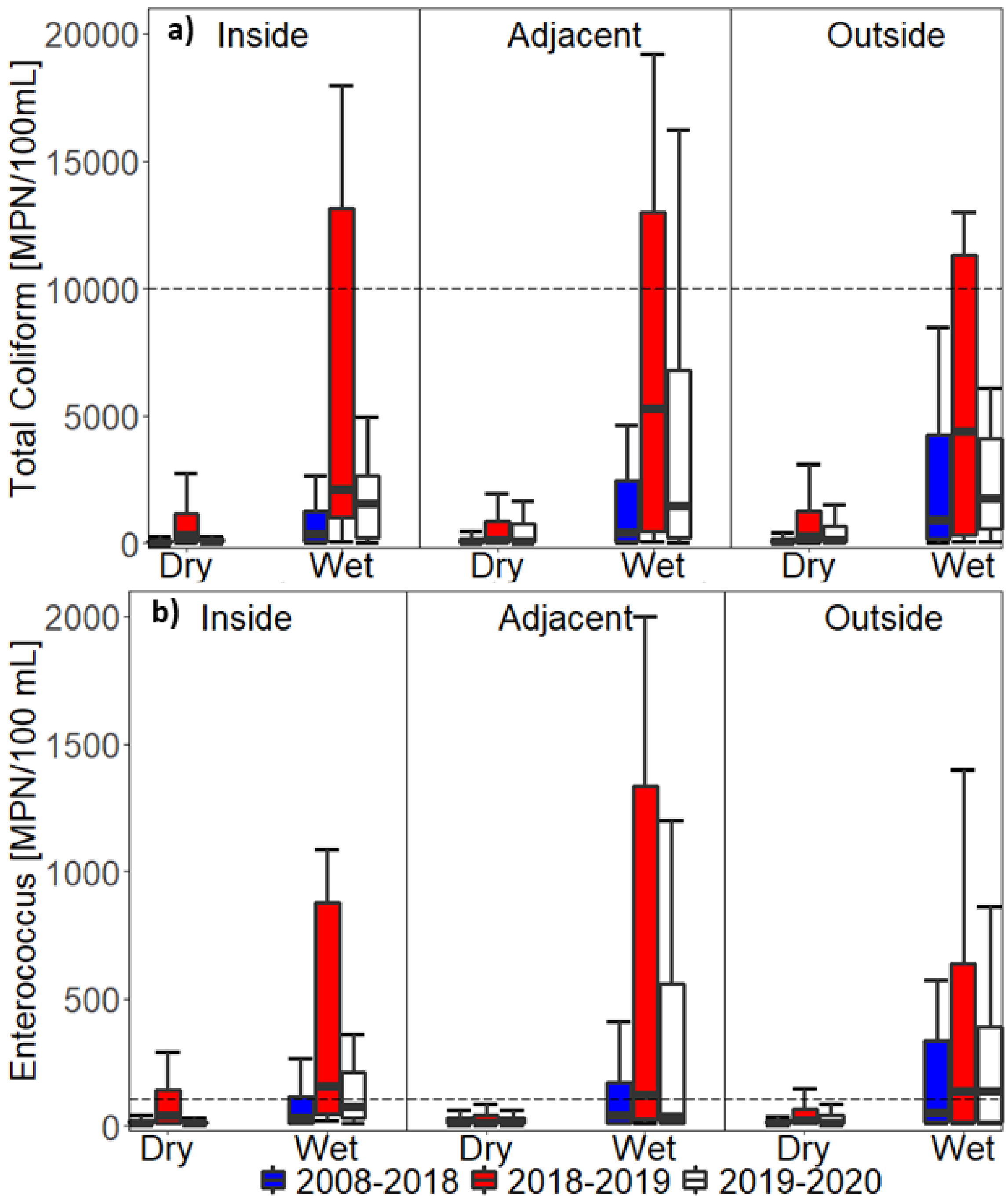

Figure S3. Boxplots of (**a**) TC and (**b**) ENT by region and weather condition. The SWRCB standards are indicated with a black dotted line. The 2018-2019 shifts in TC and ENT in the inside region occurred both during wet and dry weather.

a)

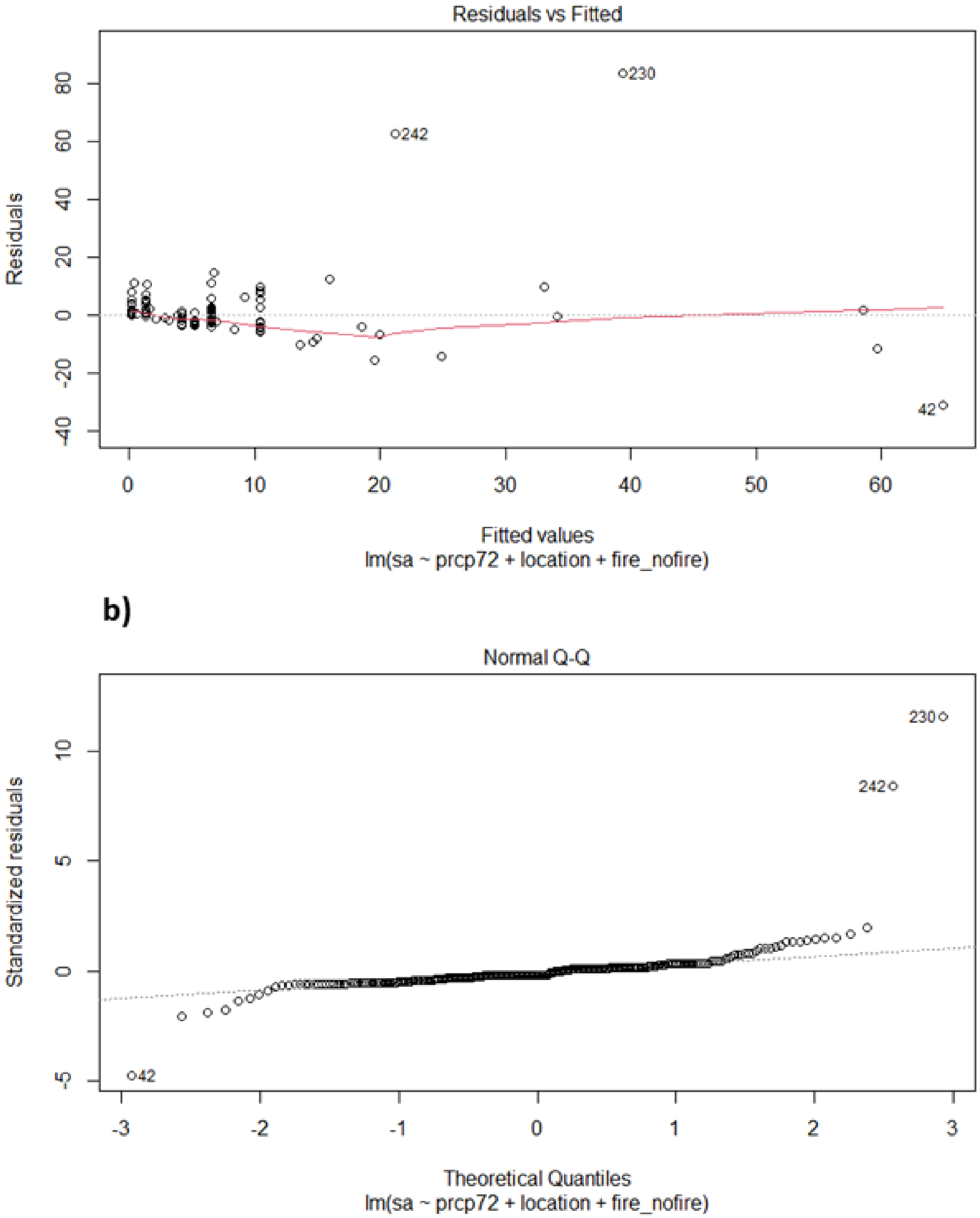

b)

Figure S4. Multiple linear regression (**a**) residuals versus fitted values plot and (**b**) residuals Q-Q plot.

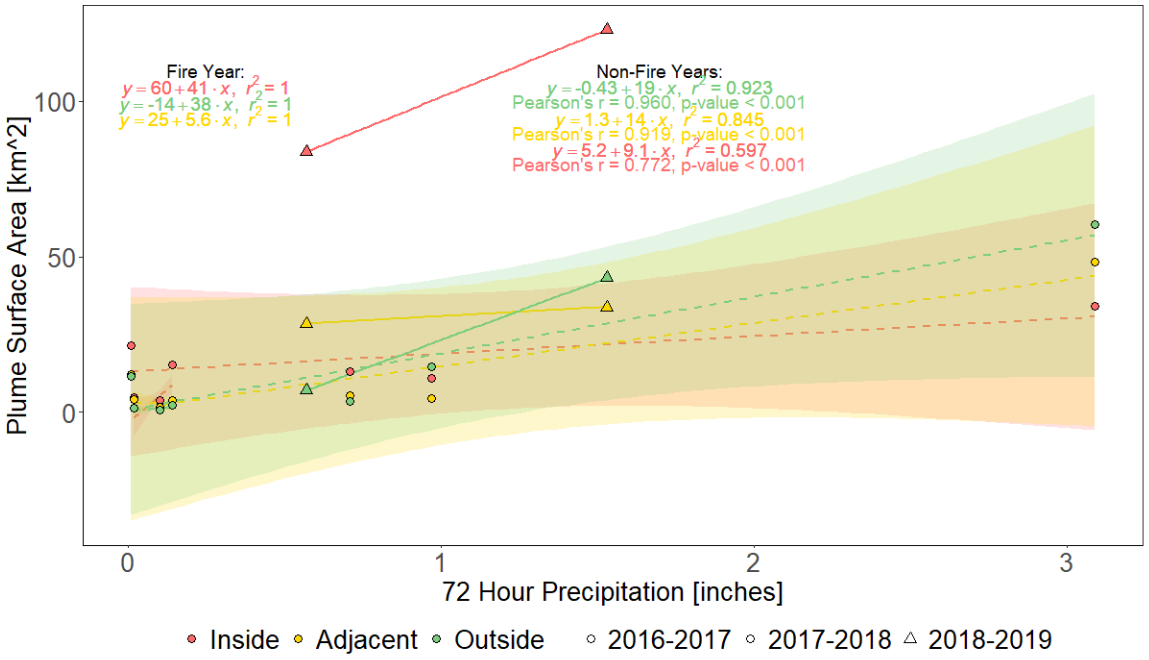

Figure S5. Linear regressions of plume surface area and 3-day precipitation totals when excluding data points with 3-day precipitation totals equal to 0.

Table S1. Boundary coordinates for the study area and each region.

	South	North	West	East
Study Area	33.9688	34.1266	-119.066	-118.520
Adjacent Left	33.9688	34.1266	-119.066	-119.018
Inside	33.9688	34.1266	-119.018	-118.681
Adjacent Right	33.9688	34.1266	-118.681	-118.582
Outside	33.9688	34.1266	-118.582	-118.520

Table S2. Sentinel-2 temporal, spatial, and spectral resolution, and swath.

	Sentinel-2
Revisit Time (days)	5
Spatial Resolution (m/pixel)	10
Swath Width (km)	290
Spectral Bands (in this study)	Band 4: Red (650 - 680 nm)
	Central Wavelength: 665 nm
	Band 8: Near-infrared (780 - 886 nm)
	Central Wavelength: 833 nm

Table S3. Systematic literature review summaries.

Search	Author	Location	Findings
“wildfire” and “turbidity”	Earl and Blinn, 2003	New Mexico	Changes in turbidity were dramatic, but short lived (<24 h). Changes in water quality occurred kilometers downstream of the actual fires.
	Rhoades et al., 2011	Colorado	Turbidity in severely burned basins (>45%) was four times greater than in lesser burned basins. Analytes remained elevated through 5 years post-fire.
	Oliver et al., 2012	California	Turbidity increased following the fire, particularly in the wetter second year. Sites (2 and 3) closest to the burned area showed greater increases.
	Murphy et al., 2012	Colorado	High intensity storms resulted in dramatic increases in turbidity at sites downstream from the burned area. Low intensity storms also led to increases in turbidity, but differences between sites upstream and downstream from the burned area remained minimal.
	Dahm et al., 2015	New Mexico	Runoff from burned areas caused turbidity peaks (to 2500 NTU) at least 50 km downstream.
	Reale et al., 2015	New Mexico	Storm events led to elevated turbidity in 1st and 2nd order streams post-fire. Less severe and nominal effects were observed in the 3rd and 4th order streams, respectively.
	Sherson et al., 2015	New Mexico	Post-fire non-monsoonal precipitation led to small increases in turbidity. Post-fire monsoonal precipitation resulted in multi-day increases in turbidity (>100 x background levels).
	Mast et al., 2016	Colorado	Post-fire high intensity rain storms caused short-term turbidity spikes (> 600 NTU).
	Lewis et al., 2018	California	Turbidity impacts of logging, harvesting, and a wildfire. Turbidity increased pre-fire at 4 sites and post-fire at 6 sites. Extreme turbidity measurements became more frequent the year following the fire.
	Thompson et al., 2018	Colorado	High turbidity spates (>1200 NTU) in late July through September 2011 were associated with loss of submerged macrophyte biomass.
	Uzun et al., 2020	California	Burned watersheds showed elevated levels in turbidity during the following two seasons. The more extensively burned watershed showed remarkable decreases in the second year.
“wildfire” and “total coliforms OR fecal coliforms OR Escherichia coli OR Enterococcus”	Valenca et al., 2020	California	Growth or persistence of E. coli in the presence of wildfire residues was less than that in the presence of unburned soil particles. Increased transport of wildfire residues did not result in increased transport of E. coli.
“wildfire,” “water quality,” and “remote sensing”	Rust et al., 2018	Western United States	Nutrient, major-ion, and metal concentrations and loading rates significantly increased within the first 5 years after fire. Particulate analytes (bounded to sediments) increased, whereas their dissolved counterparts did not. Precipitation events after fire increased particulate concentrations and loading rates.

Table S4. Wilcoxon rank sum test p-values for 2008-2018 versus 2018-2019 all weather samples. P-values <0.05, <0.01, and <0.001 are highlighted in yellow, orange, and red, respectively.

Region	Parameter	Jul	Aug	Sep	Oct	Nov	Dec	Jan	Feb	Mar	Apr	May	Jun
Inside	TC	0.931	0.062	0.350	0.552	0.984	0.002	<0.001	<0.001	<0.001	<0.001	<0.001	<0.001
	ENT	0.738	0.837	0.710	0.456	0.668	0.006	0.004	0.002	<0.001	<0.001	<0.001	<0.001
Adjacent	TC	0.026	0.007	0.001	0.566	0.003	<0.001	<0.001	<0.001	0.017	<0.001	<0.001	<0.001
	ENT	0.144	0.178	0.970	0.223	0.472	0.003	<0.001	0.004	0.876	<0.001	0.238	0.543
Outside	TC	0.785	0.266	0.800	0.247	0.973	0.051	<0.001	<0.001	<0.001	<0.001	<0.001	<0.001
	ENT	0.920	0.028	0.026	0.098	0.006	0.620	0.011	<0.001	0.004	0.002	<0.001	<0.001

Table S5. Wilcoxon rank sum test p-values for 2008-2018 versus 2018-2019 wet weather samples. P-values <0.05, <0.01, and <0.001 are highlighted in yellow, orange, and red, respectively.

Region	Parameter	Jul	Aug	Sep	Oct	Nov	Dec	Jan	Feb	Mar	Apr	May	Jun
Inside	TC	-	-	-	-	-	-	0.026	0.133	0.830	-	0.154	-
	ENT	-	-	-	-	-	-	0.051	0.197	0.133	-	0.074	-
Adjacent	TC	-	-	-	0.500	0.035	0.064	0.722	0.026	0.329	-	0.085	-
	ENT	-	-	-	0.383	0.335	0.151	0.032	0.041	0.160	-	0.591	-
Outside	TC	-	-	-	0.121	0.624	0.781	0.119	0.182	0.160	-	1.000	-
	ENT	-	-	-	0.121	0.095	0.510	0.296	0.789	0.554	-	0.698	-

Table S6. Wilcoxon rank sum test p-values for 2008-2018 versus 2018-2019 dry weather samples. P-values <0.05, <0.01, and <0.001 are highlighted in yellow, orange, and red, respectively.

Region	Parameter	Jul	Aug	Sep	Oct	Nov	Dec	Jan	Feb	Mar	Apr	May	Jun
Inside	TC	0.961	0.062	0.363	0.738	0.600	<0.001	<0.001	<0.001	<0.001	<0.001	<0.001	<0.001
	ENT	0.730	0.837	0.736	0.481	0.833	0.004	0.022	0.003	<0.001	<0.001	<0.001	<0.001
Adjacent	TC	0.023	0.007	0.001	0.362	0.004	<0.001	<0.001	0.004	0.001	<0.001	<0.001	<0.001
	ENT	0.165	0.178	0.995	0.301	0.709	0.006	0.092	0.017	0.766	<0.001	0.266	0.543
Outside	TC	0.666	0.266	0.800	0.346	0.337	0.001	<0.001	<0.001	<0.001	<0.001	<0.001	<0.001
	ENT	0.967	0.028	0.026	0.180	0.036	0.070	0.026	0.001	0.002	<0.001	<0.001	<0.001

Table S7. Pearson correlation coefficients. Moderate correlations are highlighted in yellow. P-values <0.05, <0.01, and <0.001 are marked with *, **, and ***, respectively.

Parameter	TC	ENT	SA	PRCP	PRCP72
TC	1.000				
ENT	0.545***	1.000			
SA	0.547***	0.372***	1.000		
PRCP	0.341***	0.328***	0.009	1.000	
PRCP72	0.486***	0.454***	0.634***	0.683***	1.000

Table S8. Residual normality tests. P-values <0.05, <0.01, and <0.001 are are highlighted in yellow, orange, and red, respectively.

Test	Statistic	p-value
Shapiro-Wilk	0.4879	<0.001
Kolmogorov-Smirnov	0.2499	<0.001
Cramer-von Mises	22.1935	<0.001
Anderson-Darling	30.2435	<0.001

References for Supplementary Information

1. City of Los Angeles Environmental Monitoring Division. Marine Monitoring in Santa Monica Bay: Biennial Assessment Report for the Period of January 2017 through December 2018. *Department of Public Works, LA Sanitation & Environment, Hyperion Water Reclamation Plant* , 1-228 (2019).

2. Earl, S. R. & Blinn, D. W. Effects of wildfire ash on water chemistry and biota in south-western U.S.A. streams. *Freshw. Biol.* **48**, 1015–1030 (2003).

3. Rhoades, C. C., Entwistle, D. & Butler, D. The influence of wildfire extent and severity on streamwater chemistry, sediment and temperature following the Hayman Fire, Colorado. *Int. J. Wildl. Fire* **20**, 430–442 (2011).

4. Oliver, A. A., Reuter, J. E., Heyvaert, A. C. & Dahlgren, R. A. Water quality response to the Angora Fire, Lake Tahoe, California. *Biogeochemistry* **111**, 361–376 (2012).

5. Murphy, S. F., Blaine McCleskey, R. & Writer, J. H. Effects of flow regime on stream turbidity and suspended solids after wildfire, Colorado Front Range. *IAHS-AISH Publ.* **354**, 51–58 (2012).

6. Dahm, C. N., Candelaria-Ley, R. I., Reale, C. S., Reale, J. K. & Van Horn, D. J. Extreme water quality degradation following a catastrophic forest fire. *Freshw. Biol.* **60**, 2584–2599 (2015).

7. Reale, J. K., Van Horn, D. J., Condon, K. E. & Dahm, C. N. The effects of catastrophic wildfire on water quality along a river continuum. *Freshw. Sci.* **34**, 1426–1442 (2015).

8. Sherson, L. R., Van Horn, D. J., Gomez-Velez, J. D., Crossey, L. J. & Dahm, C. N. Nutrient dynamics in an alpine headwater stream: Use of continuous water quality sensors to examine responses to wildfire and precipitation events. *Hydrol. Process.* **29**, 3193–3207 (2015).

9. Mast, M. A., Murphy, S. F., Clow, D. W., Penn, C. A. & Sexstone, G. A. Water-quality response to a high-elevation wildfire in the Colorado Front Range. *Hydrol. Process.* **30**, 1811–1823 (2016).

10. Lewis, J., Rhodes, J. J. & Bradley, C. Turbidity Responses from Timber Harvesting, Wildfire, and Post-Fire Logging in the Battle Creek Watershed, Northern California. *Environ. Manage.* **63**, 416–432 (2019).

11. Thompson, V. F., Marshall, D. L., Reale, J. K. & Dahm, C. N. The effects of a catastrophic forest fire on the biomass of submerged stream macrophytes. *Aquat. Bot.* **152**, 36–42 (2019).

12 Uzun, H. *et al.* Two years of post-wildfire impacts on dissolved organic matter, nitrogen, and precursors of disinfection by-products in California stream waters. *Water Res.* **181**, (2020).

13. Valenca, R., Ramnath, K., Dittrich, T. M., Taylor, R. E. & Mohanty, S. K. Microbial quality of surface water and subsurface soil after wildfire. *Water Res.* **175**, 115672 (2020).

14. Rust, A. J., Hogue, T. S., Saxe, S. & McCray, J. Post-fire water-quality response in the western United States. *Int. J. Wildl. Fire* **27**, 203–216 (2018).
